# Hydration attenuates incidental iliac vein stenosis detected by magnetic resonance imaging in deliberately fasted asymptomatic individuals

**DOI:** 10.1016/j.jvsv.2026.102531

**Published:** 2026-05-26

**Authors:** Kurt S. Schultz, Ronald J. Gathagan, Alexander M. Kuehne, Paula Pinto Rodriguez, Steffen Huber, Robert R. Attaran, Juan C. Perez Lozada, Britt H. Tonnessen, Edouard Aboian, Raul J. Guzman, Cassius Iyad Ochoa Chaar

**Affiliations:** a Division of Vascular Surgery and Endovascular Therapy, Department of Surgery, Yale University School of Medicine, New Haven, CT; b Department of Radiology & Biomedical Imaging, Yale University School of Medicine, New Haven, CT; c Department of Medicine, Section of Cardiology, Yale University School of Medicine, New Haven, CT

**Keywords:** Non-thrombotic iliac vein lesion, Iliac vein compression, Iliac vein stenosis, May-Thurner syndrome, Hydration status, Venous insufficiency

## Abstract

**Objective:**

Iliac vein stenosis (IVS) is observed in ≤30% of asymptomatic individuals, suggesting that it may represent a normal anatomical variant rather than a pathological condition in these cases. This study aimed to examine the effect of intravascular hydration on the degree of IVS in deliberately fasted participants without venous symptoms.

**Methods:**

This was a prospective, within-subject, analyst-blinded physiological study conducted at a single, tertiary academic hospital from August 2022 to April 2024. Adult participants without venous symptoms were instructed to fast after midnight and to present the next morning for imaging. They underwent magnetic resonance imaging (MRI) immediately before and after intravenous normal saline administration (1.0, 1.5, or 2.0 L). Two independent reviewers measured the widest and narrowest areas of the bilateral common iliac vein (CIV) and external iliac vein (EIV) using multiplanar reformatting. The primary outcome was the change in the proportion of participants with left CIV stenosis of ≥50% (defined by cross-sectional area) between prehydration and posthydration MRIs across all hydration volumes combined.

**Results:**

Among 14 participants (median age, 30 years; 50% female; 71.4% White; 85.7% non-Hispanic), the median body mass index was 26.3 (interquartile ranges [IQR], 22.9-29.8). Across all hydration volumes combined, the proportion of participants with ≥50% stenosis of the left CIV decreased from 42.9% (n = 6/14) to 7.1% (n = 1/14) after hydration (*P* = .025). The narrowest cross-sectional area of the left CIV demonstrated a substantially greater relative increase than the widest area (median, +34.5% [IQR, +21.0 to +76.3] vs median, +8.4% [IQR, +2.2 to +25.5]; *P* = .011), corresponding with a reduction in median stenosis severity from 45.7% to 36.9% (*P* = .016). Hydration similarly increased both the narrowest and the widest areas of the right CIV, the left EIV, and the right EIV. For the right CIV, the increase was more pronounced at the narrowest area (+27.1% [IQR, +9.7 to +45.0]) compared with the widest (+17.6% [IQR, +3.1 to +27.6]; *P* = .019). Neither hydration volume nor sex were associated with changes in cross-sectional area or stenosis in any iliac vein.

**Conclusions:**

In deliberately fasted individuals, intravascular hydration increased left iliac vein size and reduced the apparent degree of IVS on MRI. Considering hydration status may reduce misclassification of physiological iliac vein narrowing as pathological IVS, and validation in symptomatic patients is warranted.

**Article Highlights**

• **Type of Research:** Single-center, prospective, within-subject, analyst-blinded physiological study
• **Key Findings:** In this prospective cohort of 14 asymptomatic participants, the proportion with ≥50% left common iliac vein stenosis decreased from 42.9% on prehydration magnetic resonance imaging to 7.1% on posthydration magnetic resonance imaging, representing a statistically significant reduction.
• **Take Home Message:** Incorporating preimaging hydration protocols could reduce the misclassification of physiological iliac vein narrowing as pathological stenosis, thereby mitigating the overdiagnosis of nonthrombotic iliac vein stenosis.

Chronic venous insufficiency (CVI) affects approximately 25 million people in the United States.^1^ Iliac vein stenosis (IVS), also referred to as nonthrombotic iliac vein lesions (NIVLs), is characterized by narrowing of an iliac vein within the pelvis, most often due to extrinsic compression by an iliac artery. IVS is a recognized contributor to the pathophysiology of CVI, a debated risk factor for venous thromboembolism and a potential cause of chronic edema, pain, and venous ulceration.^2^, ^3^, ^4^, ^5^, ^6^ However, several studies have demonstrated that IVS could represent a normal anatomical variant and can be present in approximately 30% of asymptomatic individuals and ≤50% of young adults.^7^, ^8^, ^9^

Vein size is affected by changes in intraluminal pressure and intravascular volume. Previous studies have demonstrated that these factors can vary based on an individual's position, respirations, and hydration status.^10^^,^^11^ Magnetic resonance imaging (MRI) is a standard method used in evaluating patients with signs and symptoms suggestive of NIVL.^6^ Given that vein size is influenced by hydration status,^12^ a reasonable hypothesis for the high prevalence of incidental IVS in patients without symptoms of CVI is that patients are dehydrated at the time of cross-sectional imaging, especially when performed in the emergency department.^8^

Although it is known that hydration status influences vein size, there is a knowledge gap regarding how iliac vein morphology is affected by incremental intravenous fluid volumes, specifically at the site of maximal stenosis. This physiological study aimed to characterize the effect of intravascular hydration on the degree of apparent IVS in fasting individuals without venous symptoms (healthy participants). The goal of this line of inquiry was to inform the development of preimaging hydration protocols that could reduce false-positive radiographic findings that trigger unnecessary downstream clinical workups.

## Methods

### Study design

This was a prospective, within-subject, analyst-blinded physiological study conducted at a single, tertiary academic hospital from August 2022 to April 2024. Supplementary Fig 1 (online only) provides a schematic overview of the study design. Participants were instructed to fast after midnight and present the next morning at 8:00 am for imaging. They underwent MRI both before and after receiving intravenous fluid hydration over 1 hour. The fluid infusion was started immediately after the first MRI, and the second MRI was performed immediately upon completion of the infusion, after the patient voided, to minimize temporal variability in intravascular volume status. Participants were divided into three groups based on the volume of intravenous normal saline administered (1.0 L [infusion rate, 1000 mL/h], 1.5 L [infusion rate, 1500 mL/h], or 2.0 L [infusion rate, 2000 mL/h]). These volumes were selected based on the oral hydration volumes used by Behzadi et al,^12^ who demonstrated pelvic venous dilation at 1.0 to 2.0 L in healthy supine participants. These volumes were also selected to mirror standard intravenous fluid bolus quantities routinely used in clinical and perioperative settings.

### Sampling strategy

A pragmatic convenience sampling strategy was used with an intended sample size of 15 participants (5 per volume group): the first 5 enrolled participants were assigned to the 1.0-L group, the next 5 to the 1.5-L group, and the final 5 to the 2.0-L group. Participants were recruited through three methods: direct outreach to members of the Yale School of Medicine, recruitment flyers posted in public locations around New Haven, and a public webpage on the Yale website.^13^ The study was conducted in accordance with the principles of the Declaration of Helsinki. The Yale University Institutional Review Board approved the study (#2000033279), and written informed consent was obtained from all enrolled participants.

### Inclusion and exclusion criteria

Healthy participants between the ages of 18 and 60 years were eligible to participate. Healthy was defined as being without any medical issues, specifically with no lower extremity vascular-related symptoms or signs. A screening questionnaire was administered to all participants to confirm that they did not have venous insufficiency or cardiovascular, renal, or pulmonary disease. Participants who spoke English or Spanish were eligible. Participants were not excluded based on sex, gender, race, or ethnicity.

Individuals with lower-extremity vascular-related symptoms, technical limitations to acquiring an MRI, those with known claustrophobia, those who were pregnant or breastfeeding, and those with a documented history of anaphylaxis to contrast materials were ineligible. Participants with iliac vein measurements >2 standard deviations from the mean were prespecified as outliers and excluded from the primary analyses. Last, individuals with tissue expanders (eg, breast), joint replacements (eg, hip, knee), and permanent contraceptive devices were ineligible to participate to maximize safety and minimize image-quality concerns. Participants received $100 for study participation.

### Participant characteristics

Age, sex assigned at birth, self-identified race, and self-identified ethnicity were recorded for each participant. Body mass index was recorded on the day of the study. Age and body mass index were treated as continuous variables. Other demographic variables were treated as categorical.

### Imaging protocol and measurements

Two independent reviewers (A.M.K., R.J.G.) measured the bilateral common iliac veins (CIVs) and external iliac veins (EIVs) for all scans using Visage (Visage Imaging; version 7). The two reviewers were diagnostic radiology resident physicians, and they were both blinded to the study design, study objective, participant identity, and prehydration and posthydration status. A board-certified attending radiologist (S.H.), who serves as the Service Chief of Body MRI at our institution, served as a third-party adjudicator to verify the measurements.

Multiplanar reformatting was used to generate orthogonal cross-sections for measurement with manually applied double-oblique alignment along the vessel's long axis (Supplementary Fig 2, online only). Reviewers measured the cross-sectional areas of the iliac veins and recorded the corresponding long- and short-axis diameters at the site of maximal stenosis (ie, the narrowest area). For comparison, the widest cross-sectional area of the same vein, identified either proximal or distal to the stenotic segment, served as the reference to calculate the degree of stenosis (equation 1):

$$Percent\;stenosis\;\left(\%\right)=\left(1-\frac{{Area}_{stenosis}}{{Area}_{reference}}\right)\;x\;100$$

where *Area*_*stenosis*_ is the cross-sectional area of the vein at its narrowest area and *Area*_*reference*_ is the cross-sectional area of the same vein at its widest area.

The mean diameter of each vein cross-section was calculated as the average of its long- and short-axis diameters (equation 2). For consistency, posthydration measurements were taken at the same anatomical locations identified on the baseline MRI: the site of maximal stenosis and the site of the widest cross-sectional area. The anatomical etiology of IVS was not systematically recorded because it was outside the scope of this investigation.

The mean diameter of a cross-section was calculated using the following formula:

Meandiameter=(longaxis+shortaxis)/2.

### Outcomes

The primary outcome was the change in the proportion of participants with ≥50% stenosis of the left CIV, quantified by cross-sectional area, from prehydration to posthydration MRI. Because there is no universally agreed-upon cutoff for IVS, a 50% threshold was selected based on recommendations from the 2024 Consensus Statement by the VIVA Foundation, the American Venous Forum, and the American Vein and Lymphatic Society.^6^^,^^14^ The term “stenosis” refers to the radiographic finding of luminal narrowing of an iliac vein. Stenosis was recorded regardless of its etiology (eg, extrinsic compression or intrinsic venous wall abnormalities) and does not necessarily reflect hemodynamic significance. Secondary outcomes included changes in the degree of stenosis, cross-sectional areas, and diameters at the narrowest and widest areas for the left and right CIVs and EIVs.

### Statistical analysis

Inter-rater reliability for all prehydration and posthydration cross-sectional area measurements between the two reviewers was assessed using the intraclass correlation coefficient (ICC).^15^ A two-way random-effects model was used to evaluate absolute agreement. ICC values of >0.7 were considered good, >0.8 excellent, and 1.0 indicated perfect agreement.^16^ For all primary and secondary analyses, reviewer measurements were averaged to produce a single value per participant per MRI. The primary analysis compared the proportion of participants with ≥50% left CIV stenosis before and after hydration across all volume groups combined. Secondary analyses evaluated the effects of fluid volume on venous response to hydration and sex-based differences in this response. A sensitivity analysis, including any participants who met the outlier exclusion criterion, was performed to assess the robustness of the primary findings.

Categorical variables were summarized as frequencies and percentages. Between-group comparisons were performed using Fisher's exact tests. Paired categorical data were analyzed using McNemar's test. Continuous variables were reported as medians with interquartile ranges (IQRs). Between-group comparisons of continuous variables were conducted using the Mann-Whitney *U* test, and paired continuous data were analyzed using the Wilcoxon signed-rank test. For comparisons of continuous data across more than two groups, the Kruskal-Wallis test was used, with exact Wilcoxon rank-sum tests for pairwise post hoc comparisons when appropriate. Missing data were omitted from the analysis rather than being treated as a separate category.

A two-sided alpha of 0.05 was considered statistically significant, and 95% confidence intervals (CIs) were reported when applicable. Given the physiological and exploratory nature of this study, no formal power calculation or multiple-testing correction was performed. Analyses were intended to generate within-subject estimates to inform the design of future adequately powered clinical studies.

Study data were collected and managed using REDCap, an electronic data capture tool hosted at Yale University.^17^^,^^18^ Statistical analyses were performed using R version 4.3.1 (The R Foundation for Statistical Computing) and Stata 18.0 BE (StataCorp). Findings were reported according to the guidelines established by the Strengthening the Reporting of Observational Studies in Epidemiology Statement.

## Results

### Demographics

Sixteen healthy participants were enrolled. One participant, who initially denied claustrophobia, was unable to complete the protocol due to claustrophobic symptoms during the first MRI. Because the second scan was not obtained, this individual was excluded from the analytic cohort. No other data were missing. One additional participant's measurements were identified as outliers based on prespecified exclusion criteria and were therefore removed from the analytic cohort. Among the remaining 14 participants, the median age was 30 years (IQR, 29-32 years), and the sex distribution was balanced (seven females and seven males) (Table I).

**Table I:** Demographics of healthy participants and volumes of normal saline (*NS*) administered

Characteristics	Total (N = 14)	1.0 L of NS (n = 5)	1.5 L of NS (n = 4)	2.0 L of NS (n = 5)
Age, years	30.0 (29.0-32.0)	29.0 (29.0-30.0)	31.0 (30.0-32.0)	31.0 (30.0-32.0)
Sex				
Female	7 (50)	3 (60)	2 (50)	2 (40)
Male	7 (50)	2 (40)	2 (50)	3 (60)
Self-identified race				
Black	1 (7.1)	1 (20)	0 (0)	0 (0)
Asian	3 (21.4)	0 (0)	1 (25)	2 (40)
White	10 (71.4)	4 (80)	3 (75)	3 (60)
Self-identified, ethnicity				
Hispanic	2 (14.3)	1 (20)	0 (0)	1 (20)
Non-Hispanic	12 (85.7)	4 (80)	4 (100)	4 (80)
BMI	26.3 (22.2-29.8)	24.4 (24.4-24.4)	28.7 (20.3-30.0)	28.1 (22.2-29.0)

*BMI*, Body mass index.

Values are median (interquartile range) or number (%).

### Intraclass correlation

Inter-rater reliability for the left CIV was good to excellent. For the widest prehydration measurement, the ICC was 0.88 (95% CI, 0.77-0.94), indicating excellent agreement between reviewers. For the narrowest prehydration measurement, the ICC was 0.73 (95% CI, 0.52-0.86), reflecting good agreement. For the widest posthydration measurement, the ICC was 0.83 (95% CI, 0.68-0.91), indicating excellent agreement. For the narrowest posthydration measurement, the ICC was 0.77 (95% CI, 0.58-0.88), reflecting good agreement.

### Iliac vein measurements for all volume groups combined

**Fig 1:**
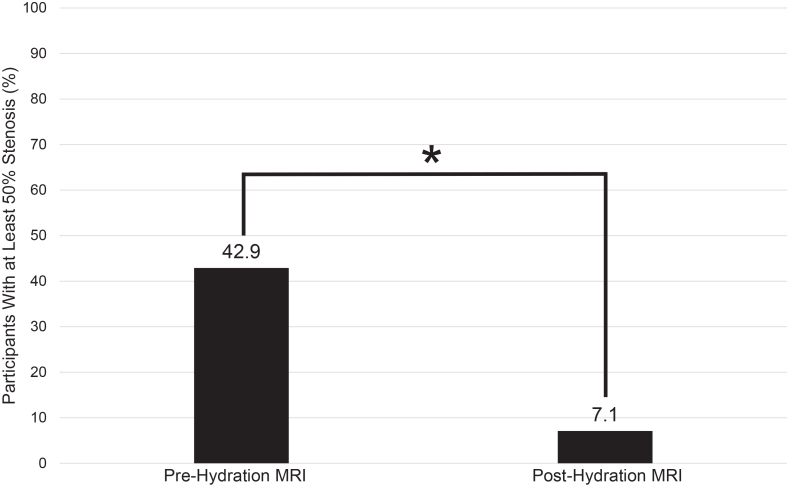
Proportion of deliberately fasted participants with at least 50% stenosis of the left common iliac vein before and after hydration. ∗*P* value < .05.

**Fig 2:**
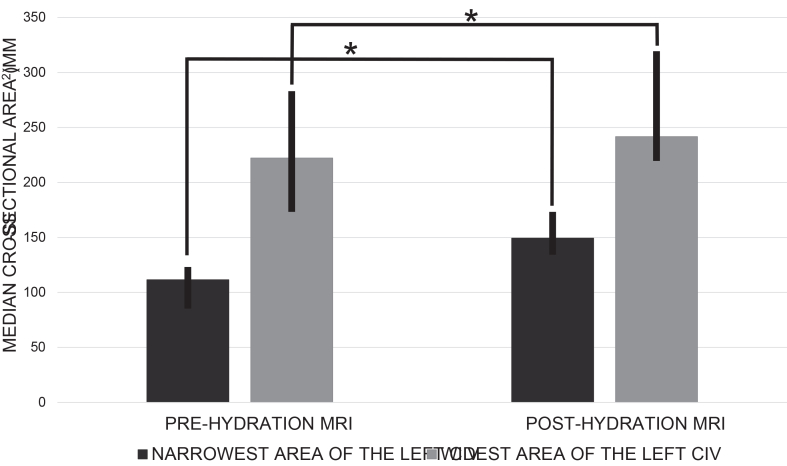
Median cross-sectional area of the narrowest and widest areas of the left common iliac vein on pre- and post-hydration magnetic resonance imaging. ∗*P* value < .05.

The proportion of participants with ≥50% stenosis of the left CIV decreased from 42.9% (n = 6/14) before hydration to 7.1% (n = 1/14) after hydration (*P* = .025) (Fig 1). Hydration caused measurable changes in the dimensions of the left CIV (Table II). At the narrowest area of the vein, the median cross-sectional area increased from 111.5 mm^2^ (IQR, 85.3-123.2 mm^2^) to 149.3 mm^2^ (IQR, 134.3-173.3 mm^2^) following hydration (*P* = .002) (Supplementary Fig 3, online only). At the widest area of the vein, the median cross-sectional area also increased from 222.2 mm^2^ (IQR, 173.3-283.1 mm^2^) to 241.7 mm^2^ (IQR, 219.6-319.4 mm^2^) following hydration (*P* = .013). The narrowest area of the left CIV increased significantly more than its widest area (relative change: median, +34.5% [IQR, +21.0% to +76.3%] vs median, +8.4% [IQR, +2.2% to +25.5%]; *P* = .011), resulting in a significant decrease in the median degree of stenosis from 45.7% to 36.9% (*P* = .016) (Fig 2).

**Table II:** Prehydration and posthydration magnetic resonance imaging (*MRI*) measurements of the left common iliac vein (CIV) in deliberately fasted participants without venous symptoms

Measurements	Prehydration MRI (n = 14)	Posthydration MRI (n = 14)	*P* value
Proportion with ≥50% stenosis	6 (42.9)	1 (7.1)	.025^a^
Degree of stenosis, %	45.7 (38.3-61.1)	36.9 (27.6-47.6)	.016^a^
Widest cross-sectional area, mm^2^	222.2 (173.3-283.1)	241.7 (219.6-319.4)	.013^a^
Long axis, mm	22.1 (19.7-23.9)	21.7 (18.9-25.8)	.925
Short axis, mm	14.1 (12.0-17.1)	15.6 (12.6-17.9)	.019^a^
Mean diameter, mm	17.5 (15.1-19.5)	18.0 (17.4-21.2)	.016^a^
Narrowest cross-sectional area, mm^2^	111.5 (85.3-123.2)	149.3 (134.3-173.3)	.002^a^
Long axis, mm	23.6 (20.8-26.5)	26.4 (24.9-29.1)	.016^a^
Short axis, mm	7.1 (5.4-9.4)	7.8 (6.3-9.3)	.096
Mean diameter, mm	12.4 (11.7-15.6)	14.3 (13.3-15.6)	.022^a^

Data are reported across all volume groups combined.

Values are number (%) or median (interquartile range).

a Significant at *P* < .05.

Hydration significantly increased both the narrowest and the widest cross-sectional areas of the right CIV (Supplementary Table I, online only), the left EIV (Supplementary Table II, online only), and the right EIV (Supplementary Table III, online only). For the right CIV, the increase was more pronounced at the narrowest area (*P* = .019), corresponding with a significant reduction in the median degree of stenosis. Although the relative expansion at the narrowest area exceeded that at the widest area for the left and right EIVs (Supplementary Table IV, online only), these differences were not statistically significant (*P* = .331 and *P* = .551, respectively) and did not translate into meaningful changes in stenosis severity.

### Prehydration and posthydration measurements, stratified by volume of normal saline

All three hydration volumes increased both the narrowest and widest cross-sectional areas of the left CIV (Supplementary Table IV, online only). Although the magnitude of relative dilation varied by volume, these differences were not statistically significant (*P* = .744 and *P* = .458 for the narrowest and widest areas, respectively). Absolute reductions in stenosis were also observed across groups (1.0-L group: median, −8.3% [IQR, −28.1% to −8.3%]; 1.5-L group: median, −4.9% [IQR, −27.3% to +8.2%]; 2.0-L group: median, −12.0% [IQR, −12.8% to −8.5%]), yet did not differ significantly by hydration volume (*P* = .831).

A similar pattern was observed in the right CIV, left EIV, and right EIV, with each volume generally increasing the cross-sectional area and reducing stenosis severity (Supplementary Table IV, online only). The only exception was a slight increase in stenosis for the right EIV in the 1.0-L group (median, +0.7% [IQR, −2.6% to +11.5%]). Across all veins examined, neither the relative change in cross-sectional area nor the absolute change in stenosis differed significantly between hydration groups.

### Prehydration and posthydration measurements for all volume groups combined, stratified by sex

There were no statistically significant sex differences in the relative changes in cross-sectional area or in the absolute change in stenosis across any of the four veins (Supplementary Table V, online only). Within-sex analyses showed a consistent pattern in which the left CIV exhibited a greater relative increase at its narrowest segment than at its widest; however, these changes did not attain statistical significance.

### Sensitivity analysis

Including the outlier in the analytic cohort yielded results consistent with the primary analysis. The proportion of participants with ≥50% stenosis of the left CIV decreased from 40.0% (n = 6/15) before hydration to 6.7% (n = 1/15) after hydration (*P* = .025). At the narrowest area of the left CIV, the median cross-sectional area increased from 109.2 mm^2^ (IQR, 81.6-123.2 mm^2^) to 149.2 mm^2^ (IQR, 122.1-173.3 mm^2^) following hydration (*P* = .002). At the widest area, the median area increased from 219.1 mm^2^ (IQR, 165.6-283.1 mm^2^) to 241.1 mm^2^ (IQR, 185.6-319.4 mm^2^; *P* = .009). The relative increase at the narrowest segment remained greater than at the widest segment (median, +32.3% [IQR, +20.3% to +76.3%] vs median, +8.7% [IQR, +2.2% to +27.9%]; *P* = .041), with a decrease in the median degree of stenosis from 44.2% to 36.9% (*P* = .069).

## Discussion

The purpose of this prospective, within-subject, analyst-blinded physiological study was to characterize the effect of intravenous hydration on iliac vein morphology in deliberately fasted individuals. Intravenous hydration increased the cross-sectional areas at both the narrowest and widest portions of the left CIV, with greater relative expansion observed at the narrowest segment. This differential response reduced the median degree of stenosis after hydration. As a result, the proportion of participants meeting commonly used imaging thresholds for significant stenosis (50% stenosis) decreased markedly after hydration, from 6 of 14 participants before hydration to 1 of 14 afterward. These findings highlight the dynamic, volume-dependent nature of iliac vein morphology and suggest that hydration status is an important determinant of image-based pelvic vein assessments in asymptomatic individuals.

Our study addresses the knowledge gap raised by the 2024 consensus statement from the VIVA Foundation, the American Venous Forum, and the American Vein and Lymphatic Society, which called for research into the role of hydration in the endovascular management of NIVLs.^6^ The concept that hydration affects venous caliber is well accepted,^19^ but few studies have systematically quantified this effect in healthy human subjects, and the data are mixed.^20^^,^^21^ Behzadi et al^12^ demonstrated that oral hydration (1.0-2.0 L) increased the CIV cross-sectional area on imaging for healthy participants in the supine position. Our study extends this work by focusing on intravenous hydration, which achieves more rapid and predictable intravascular expansion, and specifically assessing changes at the narrowest site of the vein, where pseudostenosis is likely to occur.

Pelvic venous anatomy and physiology might differ by sex, yet the evidence remains inconsistent. Both our study and that of Behzadi et al^12^ found no statistically significant sex-based differences in pelvic vein size measurements following hydration, which contrasts with findings from other research.^22^^,^^23^ A sex-based analysis was outside the primary scope of our investigation, and our study was likely underpowered to detect a substantial difference. IVS is more commonly found in younger females, and estrogen and other sex hormones increase venous relaxation and distensibility.^24^ Thus, these findings, alongside the mixed findings in the existing literature, warrant further research in adequately powered, sex-stratified cohorts to evaluate potential sex-specific differences in iliac vein compliance and hydration response.

**Fig 3:**
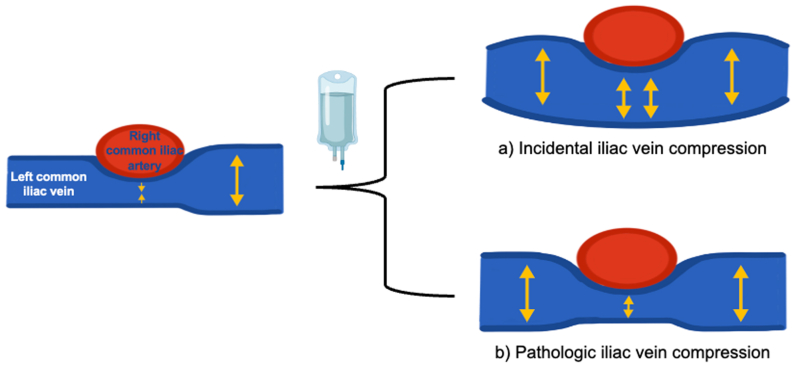
Proposed conceptual model of the impact of hydration on iliac vein size in patients with incidental iliac vein compression compared to those with pathologic compression. a) An area of physiologic venous collapse is restored to a normal caliber following a period of hydration b) An area of pathological venous collapse is not restored to a normal caliber following a period of hydration.

We propose a conceptual model (Fig 3) to explain the observed changes. We suspect that many of the stenotic segments detected on prehydration MRI represent physiological venous collapse due to fasting-induced volume depletion, rather than true pathological narrowing. Hydration appears to restore these areas to their physiological baseline, converting apparent stenosis into normal venous anatomy. Thus, fasting and dehydration might lead to the overdiagnosis of NIVLs, resulting in unnecessary venograms and stent placements.^25^ This model provides a potential explanation for the wide range of prevalence estimates for asymptomatic IVS reported in prior imaging studies.^7^, ^8^, ^9^^,^^23^ By demonstrating that the apparent stenosis prevalence decreased from 42.9% to 7.1% following controlled intravenous hydration, our study provides a mechanistic framework for reconciling these discrepant estimates from previous studies that did not standardize hydration status before imaging. The true population-level prevalence of incidental IVS likely lies somewhere between these bounds. Future studies should account for hydration status, because this variable might represent a key covariate when estimating the true prevalence of IVS. Conversely, in patients with pathological IVS, hydration could preferentially expand compliant venous segments while leaving fibrotic or stenotic segments unchanged, thereby accentuating the degree of stenosis. Previous work from our group demonstrated that stenting later in the day, a proxy for reduced hydration status, was associated with smaller stent sizes in patients with IVS,^26^ but prospective studies are needed to test the hypothesis that intravascular volume status modifies stenosis severity in symptomatic patients.

The elliptical morphology of iliac veins makes them particularly sensitive to changes in intravascular volume.^27^ This dynamic behavior is likely more pronounced in NIVLs than in fixed pathologies, such as post-thrombotic states, where fibrosis or scarring restricts venous distensibility.^6^^,^^28^ We hypothesize that, in patients with pathological compression, hydration will accentuate the relative degree of stenosis because the surrounding vein segments will expand, while the stenotic segment, which is typically inflamed and scarred, remains compressed. If validated, this mechanism could help to distinguish physiological from pathological IVS during imaging. For patients undergoing cross-sectional pelvic and lower extremity imaging, implementing a standardized preimaging hydration protocol might reduce the burden of false positives in otherwise healthy individuals, thereby reducing unnecessary downstream workups and potential overtreatment. The magnitude of stenosis reduction was similar across all three hydration volumes in our study. This finding suggests that the administration of 1.0 L of intravenous fluid might be sufficient to achieve the observed physiological effect on iliac vein caliber. Our group is conducting a subsequent study to investigate whether administering 1.0 L of fluid over 1 hour immediately before a diagnostic MRI is the optimal volume that balances diagnostic accuracy with the risk of volume overload in patients with suspected IVS.

Several limitations should be acknowledged. The use of convenience sampling introduces the potential for sampling bias. This approach likely contributed to the younger average age and lower body mass index of our cohort compared with national averages, potentially limiting generalizability. Measurement variability represents another potential limitation. To minimize this risk, two independent, blinded reviewers performed all MRI measurements, with adjudication by a third reviewer. Multiplanar reformatting with double-oblique alignment was used to obtain orthogonal cross-sections of the iliac veins, improving the accuracy and reproducibility of diameter and area assessments.^29^ This approach mitigates measurement errors from foreshortening and off-axis acquisition inherent to standard axial images. Inter-rater reliability for left CIV measurements was good to excellent, supporting the reproducibility of our measurement protocol. One participant met the outlier exclusion criteria, raising the possibility of selection bias. Sensitivity analyses including this participant demonstrated that the direction and magnitude of the hydration effects on the left CIV were consistent with the primary findings, reinforcing the robustness of the results. Last, our study protocol required overnight fasting before imaging, which is not standard for most clinical MRI findings. As such, our cohort represents a controlled model of relative hypovolemia rather than typical clinical imaging conditions, and our findings may not be directly generalizable to nonfasting patients. Future studies should evaluate the effect of hydration on iliac vein morphology in symptomatic patients to determine the broader applicability of a standardized preimaging hydration protocol.

## Conclusions

In deliberately fasted individuals, intravenous hydration increased the cross-sectional area of the left CIV, with the most pronounced expansion occurring at its narrowest segment, reducing the apparent degree of IVS. Preimaging hydration protocols could lessen the overdiagnosis of NIVLs, prevent unnecessary interventions, and enhance the diagnostic accuracy of iliac vein imaging. Future studies are needed to determine whether these physiological effects of hydration can be leveraged to improve the diagnostic accuracy of IVS in symptomatic patients in clinical practice.

## Author Contributions

Conception and design: KS, PR, SH, RA, JP, CC

Analysis and interpretation: KS, RGa, AK, SH, BT, EA, RGu, CC

Data collection: KS, RGa, AK, PR, SH, RA, JP, CC

Writing the article: KS, CC

Critical revision of the article: KS, RGa, AK, PR, SH, RA, JP, BT, EA, RGu, CC

Final approval of the article: KS, RGa, AK, PR, SH, RA, JP, BT, EA, RGu, CC

Statistical analysis: KS, CC

Obtained funding: KS, RGu, CC

Overall responsibility: CC

## Funding

This study was supported by the 2023 AVF-JOBST Clinical Research Grant. The funder had no role in study design, data collection, analysis, or interpretation, manuscript preparation, or the decision to submit for publication.

## Disclosures

K.S. has received research support from the American Venous Forum. C.I.O.C. is a consultant for EnVVeno Medical, has IP of patent U.S.S.N. 10,524,89, and has received research support from the Yale Department of Surgery, Society for Vascular Surgery, American Venous Forum, CT Innovation, Vascular Study Group of New England, National Institutes of Health, Boston Scientific, Medtronic, EnVVeno Medical, and Inari Medical.
