# Supplementary material for: Hydration attenuates incidental iliac vein stenosis detected by magnetic resonance imaging in deliberately fasted asymptomatic individuals
Source: J Vasc Surg Venous Lymphat Disord. 2026 May 26;14(5):102531. doi: 10.1016/j.jvsv.2026.102531 (PMC13325909; doi:10.1016/j.jvsv.2026.102531)
Supplement: Supplementary Figures 1 and 2 (online only) [file mmc1.docx]

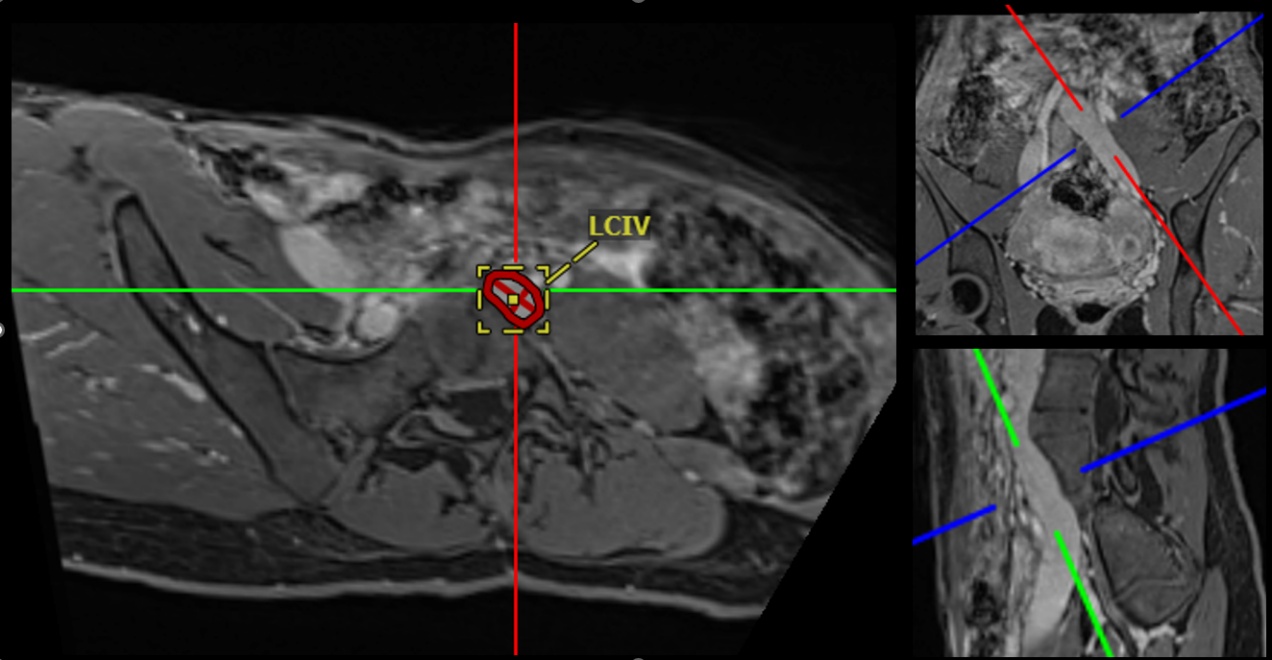


**eFigure 1. Static depiction of multiplanar reformatting of the left common iliac vein at its widest cross-sectional area, shown in three oblique planes.**

This representative magnetic resonance imaging is from a healthy participant after one hour of intravenous hydration. Multiplanar reformatting, using a double-oblique alignment along the vessel’s long axis, provides true-to-anatomy orthogonal views that improve accuracy and reproducibility of venous diameter and area measurements.


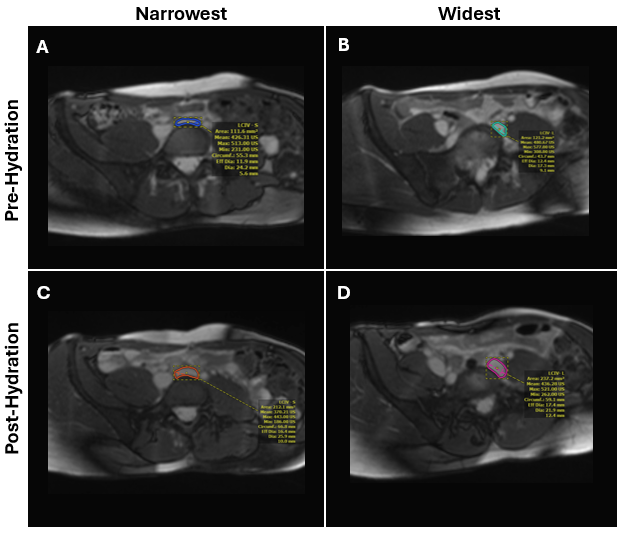


**eFigure 2. Measurements of the left common iliac vein on pre- and post-hydration magnetic resonance imaging in a 31-year-old female participant without venous symptoms.**

a) Narrowest cross-sectional area on a pre-hydration MRI. b) Widest cross-sectional area on the same pre-hydration MRI. c) Narrowest cross-sectional area on a post-hydration MRI. d) Widest cross-sectional area on the same post-hydration MRI. The colored circles outline the two areas of interest of the left CIV on pre- and post-hydration imaging**.** *Abbreviations:* *MRI, magnetic resonance imaging; CIV, common iliac vein.*
