# Supplementary material for: Hydration attenuates incidental iliac vein stenosis detected by magnetic resonance imaging in deliberately fasted asymptomatic individuals
Source: J Vasc Surg Venous Lymphat Disord. 2026 May 26;14(5):102531. doi: 10.1016/j.jvsv.2026.102531 (PMC13325909; doi:10.1016/j.jvsv.2026.102531)
Supplement: Supplementary Methods (online only) [file mmc2.docx]

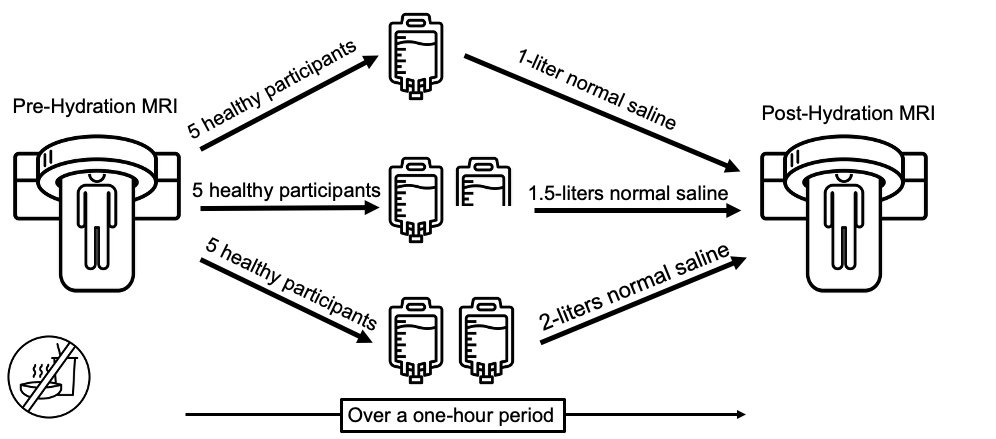


**eMethods.** Schematic representation of the study design. Participants were instructed to fast at midnight and present the next morning at 8:00 AM for imaging. The fluid infusion was started immediately following the first MRI, and the second MRI was performed immediately upon completion of the infusion, after the patient voided, to minimize temporal variability in intravascular volume status. The measurements for one participant in the 1.5-liter group met the prespecified exclusion criteria, and this participant was removed from the analytic cohort, leaving 14 participants for subsequent analyses.
