# Supplementary material for: Hydration attenuates incidental iliac vein stenosis detected by magnetic resonance imaging in deliberately fasted asymptomatic individuals
Source: J Vasc Surg Venous Lymphat Disord. 2026 May 26;14(5):102531. doi: 10.1016/j.jvsv.2026.102531 (PMC13325909; doi:10.1016/j.jvsv.2026.102531)
Supplement: Supplementary Tables I-V (online only) [file mmc3.docx]

**eTable 1. Pre- and post-hydration magnetic resonance imaging measurements of the right common iliac vein in deliberately fasted participants without venous symptoms.**

| **Measurements, median (IQR)** | **Pre-Hydration MRI**  **(n=14)** | **Post-Hydration MRI**  **(n=14)** | **p-value** |
| --- | --- | --- | --- |
| Proportion with $\geq$ 50% stenosis, n (%) | 0 (0.0) | 0 (0.0) | - |
| Degree of stenosis, % | 35.3 (26.0-39.1) | 29.9 (23.3-34.7) | 0.041 |
| Widest cross-sectional area, mm^2^ | 166.3 (119.1-238.4) | 197.0 (167.4-244.7) | 0.019 |
| Long axis, mm | 18.3 (16.8-21.0) | 19.6 (17.5-19.9) | 0.096 |
| Short axis, mm | 13.2 (10.5-15.8) | 14.6 (11.6-16.5) | 0.008 |
| Mean diameter, mm | 15.3 (13.6-18.3) | 16.8 (14.7-18.5) | 0.011 |
| Narrowest cross-sectional area, mm^2^ | 116.7 (83.9-136.8) | 148.5 (125.6-161.5) | 0.016 |
| Long axis, mm | 15.2 (13.9-17.2) | 17.4 (14.8-19.0) | 0.026 |
| Short axis, mm | 10.5 (8.2-12.4) | 12.1 (10.0-13.5) | 0.008 |
| Mean diameter, mm | 12.9 (10.7-14.2) | 14.8 (12.9-15.5) | 0.009 |

Data are reported across all volume groups combined. *Abbreviations: MRI, magnetic resonance imaging; IQR, interquartile range; mm^2^, square millimeters.*

**eTable 2. Pre- and post-hydration magnetic resonance imaging measurements of the left external iliac vein in deliberately fasted participants without venous symptoms.**

| **Measurements, median (IQR)** | **Pre-Hydration MRI**  **(n=14)** | **Post-Hydration MRI**  **(n=14)** | **p-value** |
| --- | --- | --- | --- |
| Proportion with $\geq$ 50% stenosis, n (%) | 1 (7.1) | 0 (0.0) | 0.317 |
| Degree of stenosis, % | 38.0 (26.4-44.4) | 36.0 (22.7-43.1) | 0.397 |
| Widest cross-sectional area, mm^2^ | 168.7 (133.5-257.9) | 204.5 (144.4-281.4) | 0.005 |
| Long axis, mm | 17.4 (15.2-20.4) | 19.4 (17.1-22.9) | 0.009 |
| Short axis, mm | 13.3 (12.3-16.4) | 13.6 (12.3-17.0) | 0.109 |
| Mean diameter, mm | 15.4 (13.6-18.7) | 16.8 (14.1-19.9) | 0.005 |
| Narrowest cross-sectional area, mm^2^ | 117.4 (100.9-156.6) | 136.7 (104.3-182.2) | 0.026 |
| Long axis, mm | 16.4 (14.9-18.2) | 17.1 (15.7-19.3) | 0.177 |
| Short axis, mm | 9.4 (8.3-11.1) | 10.5 (9.8-12.1) | 0.010 |
| Mean diameter, mm | 12.4 (11.4-14.3) | 13.6 (12.4-15.4) | 0.013 |

Data are reported across all volume groups combined. *Abbreviations: MRI, magnetic resonance imaging; IQR, interquartile range; mm^2^, square millimeters.*

| **Measurements, median (IQR)** | **Pre-Hydration MRI**  **(n=14)** | **Post-Hydration MRI**  **(n=14)** | **p-value** |
| --- | --- | --- | --- |
| Proportion with $\geq$ 50% stenosis, n (%) | 3 (21.4) | 2 (14.3) | 0.317 |
| Degree of stenosis, % | 39.0 (26.7-48.5) | 34.6 (29.3-49.2) | 0.683 |
| Widest cross-sectional area, mm^2^ | 156.3 (135.5-200.4) | 192.1 (153.4-260.4) | 0.001 |
| Long axis, mm | 16.4 (15.6-20.1) | 19.0 (17.0-21.0) | 0.038 |
| Short axis, mm | 13.0 (11.5-14.0) | 14.1 (12.3-16.3) | 0.002 |
| Mean diameter, mm | 14.5 (13.4-16.6) | 16.6 (14.4-18.2) | 0.001 |
| Narrowest cross-sectional area, mm^2^ | 94.5 (69.7-132.4) | 113.0 (93.5-161.6) | 0.002 |
| Long axis, mm | 15.2 (13.5-17.0) | 16.8 (13.4-17.5) | 0.048 |
| Short axis, mm | 8.5 (7.7-9.7) | 9.8 (8.0-12.7) | 0.016 |
| Mean diameter, mm | 11.3 (9.7-12.9) | 12.1 (10.9-14.8) | 0.004 |

**eTable 3. Pre- and post-hydration magnetic resonance imaging measurements of the right external iliac vein in deliberately fasted participants without venous symptoms.**

Data are reported across all volume groups combined. *Abbreviations: MRI, magnetic resonance imaging; IQR, interquartile range; mm^2^, square millimeters.*

**eTable 4. Changes in magnetic resonance imaging measurements of the iliac veins following hydration, stratified by volume of normal saline received.**

| **Measurements, % - median (IQR)** | **Overall**  **(n=14)** | **1 liter of NS**  **(n=5)** | **1.5 liters of NS**  **(n=4)** | **2 liters of NS**  **(n=5)** | **p-value** |
| --- | --- | --- | --- | --- | --- |
| Left common iliac vein |  |  |  |  |  |
| Relative change in widest CSA | +8.4 (+2.2 to +25.5) | +8.7 (+4.3 to +16.9) | +3.3 (-9.6 to +18.1) | +12.8 (+7.6 to +32.1) | 0.458 |
| Relative change in narrowest CSA | +34.5 (+21.0 to +76.3) | +23.8 (+23.7 to +76.3) | +17.9 (-1.6 to +74.8) | +43.2 (+32.3 to +44.0) | 0.744 |
| Absolute change in degree of stenosis | -9.4 (-14.2 to -4.9) | -8.3 (-28.1 to -8.3) | -4.9 (-27.3 to +8.2) | -12.0 (-12.8 to -8.5) | 0.831 |
| Right common iliac vein |  |  |  |  |  |
| Relative change in widest CSA | +17.6 (+3.1 to +27.6) | +18.4 (+15.1 to +20.7) | +13.9 (-10.0 to +26.4) | +16.8 (+5.0 to +44.9) | 0.779 |
| Relative change in narrowest CSA | +27.1 (+9.7 to +45.0) | +23.2 (+13.1 to +45.0) | +27.4 (-2.7 to +35.2) | +30.4 (+0.5 to +70.1) | 0.914 |
| Absolute change in degree of stenosis | -5.0 (-8.2 to -1.4) | -6.6 (-7.0 to -2.6) | -4.8 (-9.4 to +2.1) | -3.4 (-8.2 to -1.4) | 0.990 |
| Left external iliac vein |  |  |  |  |  |
| Relative change in widest CSA | +8.3 (+5.3 to +19.1) | +6.0 (-4.4 to +6.5) | +18.6 (+5.8 to +30.9) | +12.8 (+12.2 to +19.1) | 0.154 |
| Relative change in narrowest CSA | +13.9 (-4.8 to +26.2) | +14.5 (-4.8 to +15.8) | +21.7 (+3.1 to +34.2) | +13.3 (+4.0 to +25.0) | 0.831 |
| Absolute change in degree of stenosis | -3.1 (-8.0 to +6.2) | -3.9 (-8.0 to -2.5) | -3.5 (-8.2 to +8.6) | -0.6 (-2.9 to +6.2) | 0.825 |
| Right external iliac vein |  |  |  |  |  |
| Relative change in widest CSA | +13.3 (+6.1 to +29.2) | +13.3 (+13.3 to +44.5) | +7.8 (+4.5 to +15.0) | +19.4 (+12.9 to +29.2) | 0.236 |
| Relative change in narrowest CSA | +14.6 (+6.7 to +46.6) | +11.6 (+11.3 to +17.5) | +23.6 (+0.3 to +48.0) | +26.8 (+11.2 to +37.9) | 0.835 |
| Absolute change in degree of stenosis | -1.4 (-5.4 to +6.7) | +0.7 (-2.6 to +11.5) | -6.5 (-16.6 to +4.3) | -3.8 (-5.4 to -0.2) | 0.367 |

Relative change in cross-sectional areas was calculated as the within-subject difference between the post-hydration and pre-hydration measurements divided by the pre-hydration measurement. Absolute change in percent stenosis was calculated as the post-hydration stenosis minus the pre-hydration stenosis. Negative values denote a decrease from pre- and post-hydration measurements. *Abbreviations: NS, normal saline; CSA, cross-sectional area; IQR, interquartile range.*

**eTable 5. Changes in magnetic resonance imaging measurements of the iliac veins following hydration, stratified by sex.**

| **Measurements, % - median (IQR)** | **Female sex**  **(n=7)** | **Male sex**  **(n=7)** | **p-value** |
| --- | --- | --- | --- |
| Left common iliac vein |  |  |  |
| Relative change in widest cross-sectional area | +12.8 (+2.2 to +27.9) | +8.2 (-0.4 to +25.5) | 0.749 |
| Relative change in narrowest cross-sectional area | +76.3 (+23.7 to +84.0) | +23.8 (+20.3 to +43.2) | 0.142 |
| Absolute change in degree of stenosis | -14.2 (-31.3 to -8.2) | -8.3 (-12.0 to +0.5) | 0.180 |
| Right common iliac vein |  |  |  |
| Relative change in widest cross-sectional area | +25.2 (+18.4 to +44.9) | +5.0 (+2.6 to +16.8) | 0.110 |
| Relative change in narrowest cross-sectional area | +30.9 (+9.7 to +70.1) | +23.8 (+0.5 to +45.0) | 0.655 |
| Absolute change in degree of stenosis | -2.6 (-7.4 to -1.4) | -6.6 (-11.3 to +2.6) | 0.565 |
| Left external iliac vein |  |  |  |
| Relative change in widest cross-sectional area | +12.8 (+6.0 to +30.9) | +6.4 (-2.9 to +12.2) | 0.225 |
| Relative change in narrowest cross-sectional area | +25.0 (-5.9 to +37.1) | +13.3 (+4.0 to +15.8) | 0.565 |
| Absolute change in degree of stenosis | -3.4 (-8.0 to +11.7) | -2.5 (-11.9 to +6.2) | 0.949 |
| Right external iliac vein |  |  |  |
| Relative change in widest cross-sectional area | +19.8 (+6.1 to +44.5) | +13.3 (+6.0 to +19.4) | 0.482 |
| Relative change in narrowest cross-sectional area | +37.9 (+11.2 to +49.5) | +11.6 (+0.6 to +26.8) | 0.482 |
| Absolute change in degree of stenosis | -4.1 (-14.7 to +11.5) | -0.2 (-3.8 to +1.8) | 0.749 |

Relative change in cross-sectional areas was calculated as the within-subject difference between the post-hydration and pre-hydration measurements divided by the pre-hydration measurement. Absolute change in percent stenosis was calculated as the post-hydration stenosis minus the pre-hydration stenosis. Negative values denote a decrease from pre- and post-hydration measurements. Data are reported across all volume groups combined. *Abbreviations: IQR, interquartile range.*
